# Capsule Typing of *Haemophilus influenzae* by Matrix-Assisted Laser Desorption/Ionization Time-of-Flight Mass Spectrometry^1^

**DOI:** 10.3201/eid2403.170459

**Published:** 2018-03

**Authors:** Viktor Månsson, Janet R. Gilsdorf, Gunnar Kahlmeter, Mogens Kilian, J. Simon Kroll, Kristian Riesbeck, Fredrik Resman

**Affiliations:** Lund University, Malmö, Sweden (V. Månsson, K. Riesbeck, F. Resman);; University of Michigan, Ann Arbor, Michigan, USA (J.R. Gilsdorf); Växjö Hospital, Växjö, Sweden (G. Kahlmeter);; Aarhus University, Aarhus, Denmark (M. Kilian);; Imperial College London, London, UK (J.S. Kroll)

**Keywords:** Haemophilus influenzae, bacteria, bacterial typing, serotyping, bacterial capsules, capsule typing, respiratory infections, vaccines, matrix-assisted laser desorption/ionization mass spectrometry, MALDI-TOF mass spectrometry, epidemiology, Sweden

## Abstract

Encapsulated *Haemophilus influenzae* strains belong to type-specific genetic lineages. Reliable capsule typing requires PCR, but a more efficient method would be useful. We evaluated capsule typing by using matrix-assisted laser desorption/ionization time-of-flight (MALDI-TOF) mass spectrometry. Isolates of all capsule types (a−f and nontypeable; n = 258) and isogenic capsule transformants (types a−d) were investigated. Principal component and biomarker analyses of mass spectra showed clustering, and mass peaks correlated with capsule type-specific genetic lineages. We used 31 selected isolates to construct a capsule typing database. Validation with the remaining isolates (n = 227) showed 100% sensitivity and 92.2% specificity for encapsulated strains (a−f; n = 61). Blinded validation of a supplemented database (n = 50) using clinical isolates (n = 126) showed 100% sensitivity and 100% specificity for encapsulated strains (b, e, and f; n = 28). MALDI-TOF mass spectrometry is an accurate method for capsule typing of *H. influenzae.*

*Haemophilus influenzae* is subdivided into encapsulated strains, which express different serotypes of capsular polysaccharide (designated types a–f), and nonencapsulated strains, which are designated nontypeable *H. influenzae* (NTHi) (*1*). Since the introduction of conjugate vaccines against *H. influenzae* type b (Hib), a common cause of meningitis, epiglottitis, and sepsis in small children, the epidemiology of invasive *H. influenzae* disease has changed dramatically, with an increase in the diversity of serotypes responsible for illness.

Although the incidence of Hib disease has decreased in countries implementing childhood vaccination (*2*), invasive disease caused by NTHi has become more prominent during the same period, especially among newborns and the elderly (*3**–**6*). In the postvaccination era, increasing incidences and outbreaks of invasive *H. influenzae* type a (Hia) infections have been reported in South and North America (*7**–**10*), particularly among the indigenous populations in Canada and the United States (*7**,**8**,**10*). Studies have also suggested an increase in cases of invasive *H. influenzae* type e (Hie) and type f (Hif) disease (*4**,**11**,**12*). Hib vaccine failures have been described (*13*), and omission of booster dose(s) appears to result in a rapidly increased incidence of invasive Hib disease (*14**,**15*), suggesting continued circulation of Hib isolates in the community. Globally, one third of eligible children still do not receive adequate vaccination (*16*).

Encapsulated *H. influenzae* strains are generally genetically clonal. This finding was first demonstrated by multilocus enzyme electrophoresis, by which encapsulated isolates could be separated into different genetic lineages that correspond to different capsule types (*17*). This clonal population structure has been confirmed by multilocus sequence typing (MLST), which assigns isolates to different sequence types (STs), although some differences have been observed in the organization of different lineages (*18*). There are 3 known major genetic groups of Hia and Hib (*18**,**19*). Two genetic groups of Hia (related to ST21 and ST23) account for most Hia isolates in the MLST database (*20*). For Hib, ST6-related isolates account for most cases (*17**,**18*), whereas the second most common genetic group is related to ST222 (*18**,**21*). There is 1 known lineage each for serotypes c through f (*18**,**19*). In contrast, NTHi are genetically heterogenous (*19*).

Capsule typing of *H. influenzae* has traditionally been performed by using slide agglutination with antisera (conventional serotyping), but incorrect results are common, and specificity for encapsulated isolates is low (*22**,**23*). Determination of presence of the capsule gene complex (*bexA* or *bexB*) by PCR, followed by type-specific *cap* a–f PCRs has excellent sensitivity and specificity but is laborious and time-consuming (*24**–**27*). Because of limitations of current typing methods, typing might be delayed or not performed in clinical practice. However, rapidly obtained information on capsule type is still of interest for the treating clinician (*5*) and, in particular, for monitoring of capsule type distribution and effectiveness of Hib vaccination programs, especially with respect to invasive disease.

Matrix-assisted laser desorption/ionization time-of-flight (MALDI-TOF) mass spectrometry is commonly used to identify bacterial and fungal species, including *H. influenza*e, by analyzing the composition of ribosomal proteins in a sample. It is a rapid and convenient method and has a low cost per sample (*28*). Recently, we have shown that MALDI-TOF mass spectrometry can separate Hib from non-b *H. influenzae* (*29*). In this study, we examined the capacity of MALDI-TOF mass spectrometry to perform full capsule typing of *H. influenzae*. This method would be valuable for first-line diagnostics of *H. influenzae* to identify patients at risk for immunodeficiency or anatomic cerebrospinal fluid space defect, and to detect rapidly outbreaks caused by specific capsule types. It would also increase time and cost effectiveness of surveillance of *H. influenzae* epidemiology and Hib vaccination efficacy.

## Materials and Methods

### Bacterial Isolates

**Figure 1:**
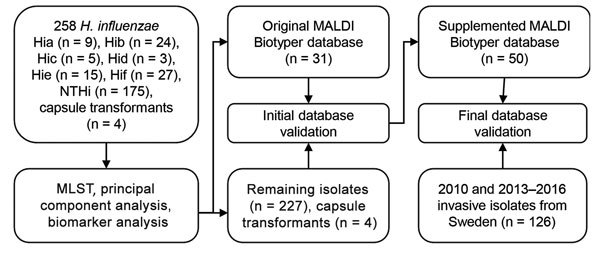
Culture collections and methods used in this study for capsule typing of *Haemophilus influenzae* by MALDI-TOF mass spectrometry. An evaluation set of *H. influenzae* isolates of all capsule types from diverse geographic origins and time periods and isogenic capsule transformants (*30*) were used to investigate capsule type-specific differences in MALDI-TOF mass spectra. MLST was used to ensure adequate coverage of different genetic lineages of encapsulated *H. influenzae*. Reference isolates from the evaluation set (encapsulated and nonencapsulated) were selected to construct a new typing database in MALDI Biotyper. This database was tested with the remaining isolates in the set, and misclassified isolates were added to the database. The final supplemented database was blindly validated with a second culture collection that consisted of clinical invasive isolates. Hia, *H. influenzae* type a; Hib, *H. influenzae* type b; Hic, *H. influenzae* type c; Hid, *H. influenzae* type d; Hie, *H. influenzae* type e; Hif, *H. influenzae* type f; MALDI-TOF, matrix-assisted laser desorption/ionization time-of-flight; MLST, multilocus sequence typing; NTHi, nontypeable *H. influenzae.*

We used 2 culture collections in this study (Figure 1). The first collection was an evaluation set of isolates used to construct a coherent reference database and was composed of 258 *H. influenzae* strains. It included isolates from 3 major clinical laboratories in Sweden (Malmö/Lund, Gothenburg, and Stockholm) obtained in 1997–2011 but also a wide range of international strains from different countries, continents, and time periods (n = 41; Technical Appendix Table). In addition, we included 4 isogenic capsule-transformed strains of types a (Rb^–^/a^+^:02), b (Rb^+^:02), c (Rb^–^/c^+^:02), and d (Rb^–^/d^+^:02) (*30*) in the study. These strains originate from strain Rd, a capsule-deficient type d strain (*31*). For validation of the new MALDI-TOF mass spectrometry typing method, we used a second collection composed of 126 bloodstream and cerebrospinal fluid *H. influenzae* isolates obtained in Sweden during 2010 and 2013–2016. All isolates were identified as *H. influenzae* by using standard laboratory taxonomy techniques and were grown on chocolate agar plates overnight (18–24 h) in a humid atmosphere at 37°C containing 5% CO_2_ before any experiments were conducted.

### PCR for Capsule Typing and MLST

We prepared DNA by adding a few colonies of bacteria to distilled water. After heating at 98°C for 10 min, we centrifuged each sample at 16,000 × *g* for 5–10 min and collected the supernatant. In a few instances, we extracted DNA by using the GenElute Bacterial Genomic DNA Kit (Sigma-Aldrich, St. Louis, MO, USA) according to the manufacturer’s instructions. We performed capsule typing by PCR using *bexB* and type-specific *cap* primers for all isolates as described (*24**–**26*).

We performed PCR for MLST genes as described (*18*) and sequenced the resulting PCR products by using the forward primer and, if necessary for adequate sequence quality, the reverse primer. We trimmed and edited sequences before concatenation (total length 3,057 bp). We deposited MLST nucleotide sequences in GenBank (accession nos. MG550316–MG550889). Some isolates had been previously typed by MLST. In these instances, we retrieved MLST data from the MLST database (*20*).

### Analysis of MLST Data

We determined sequence types by using the MLST database. We aligned concatenated sequences in Geneious 9.1.8 (Biomatters, Auckland, New Zealand) and used the PAUP* 4.0a158 plug-in (http://phylosolutions.com/paup-test/) to construct a maximum-likelihood phylogenetic tree. The best fitting model was estimated to be the generalized time-reversible model including invariant sites and gamma distribution by using the Akaike information criterion in jModelTest 2.1.10 (*32**,**33*). We visualized the resulting tree by using FigTree 1.4.3 (http://tree.bio.ed.ac.uk/software/figtree/). All isolate and ST information has been submitted to the MLST database.

### Acquisition of MALDI-TOF Mass Spectrometry Data

We acquired mass spectra by using a Microflex LT MALDI-TOF mass spectrometry system (Bruker Daltonics, Bremen, Germany), with default settings as described (*29*). We prepared all isolates for acquisition of spectra by using the ethanol–formic acid procedure described by the instrument manufacturer. We spotted isolates on 2 spots and analyzed each spot 3 times, resulting in 6 spectra/isolate. Isolates in the reference database were spotted on 8 spots, resulting in 24 spectra, before being added to the database.

### Analysis of MALDI-TOF Mass Spectrometry Data

In Mass-Up 1.0.13 (*34*), we preprocessed and analyzed raw spectra of all isolates in the evaluation set (n = 258) and capsule transformants (n = 4) by using the integrated MALDIquant analysis package for R (http://strimmerlab.org/software/maldiquant/). We performed preprocessing with intensity transformation (square root), smoothing (Savitzky–Golay), baseline correction (Top-Hat), and intensity standardization (total ion current). We performed peak detection with a signal-to-noise ratio of 2, a half window size of 50, and no minimum peak intensity. We calculated a consensus spectrum for each isolate with a peak tolerance of 0.002 and percentage of presence of 60%. For principal component analysis (PCA) (*35*) and biomarker analysis, we performed intersample matching with a peak tolerance of 0.002. PCA was performed with default settings (maximum number of components = −1 and 0.95 of the total variance covered). In the biomarker analysis, we calculated a p value for each peak by using the randomization test of independence.

### Construction and Validation of a MALDI Biotyper Database for Capsule Typing

We used selected isolates from the evaluation set to create main spectra (MSPs) for a new MALDI Biotyper 4.1 database (Bruker Daltonics) (Figure 1). These reference isolates were selected to represent all capsule types and genetic lineages. NTHi strains were selected with the aim of including isolates of all known genetic clades (*19*). Spectra of reference isolates were controlled by using FlexAnalysis (Bruker Daltonics). We performed smoothing (Savitzky-Golay) and baseline correction (Top-Hat) and excluded spectra with outlier appearance (lacking or having an extra peak) and low quality (peaks outside a 500 ppm range). If <20 spectra remained after the control, new spectra for that specific isolate were obtained. We used default settings for spectra preprocessing, MSP creation, and identification as described (*29*).

We used isolates in the evaluation set not selected as reference isolates for initial validation of the database (Figure 1). Because all isolates were *H. influenzae*, high score values (>2.0) were expected. Thus, we classified each spectrum according to the top matching MSP in the new database. For isolate classification, >5/6 spectra classified to the same type (a–f or NTHi) were required. If <4/6 spectra were classified to the same type, the isolate was classified as inconclusive. To improve the specificity of the typing method, considering the known heterogeneity of NTHi, we supplemented the capsule typing database with NTHi isolates not correctly classified in the initial validation until all isolates in the evaluation set not included in the database were correctly classified on every single spectrum. Finally, we blindly validated the supplemented database by using MALDI-TOF mass spectrometry classification of invasive isolates (n = 126) obtained during 2010 and 2013–2016 and calculated sensitivity and specificity by using PCR typing as the standard (Figure 1).

## Results

### Genetic Lineages of Encapsulated *H. influenzae* in the Evaluation Set

**Figure 2:**
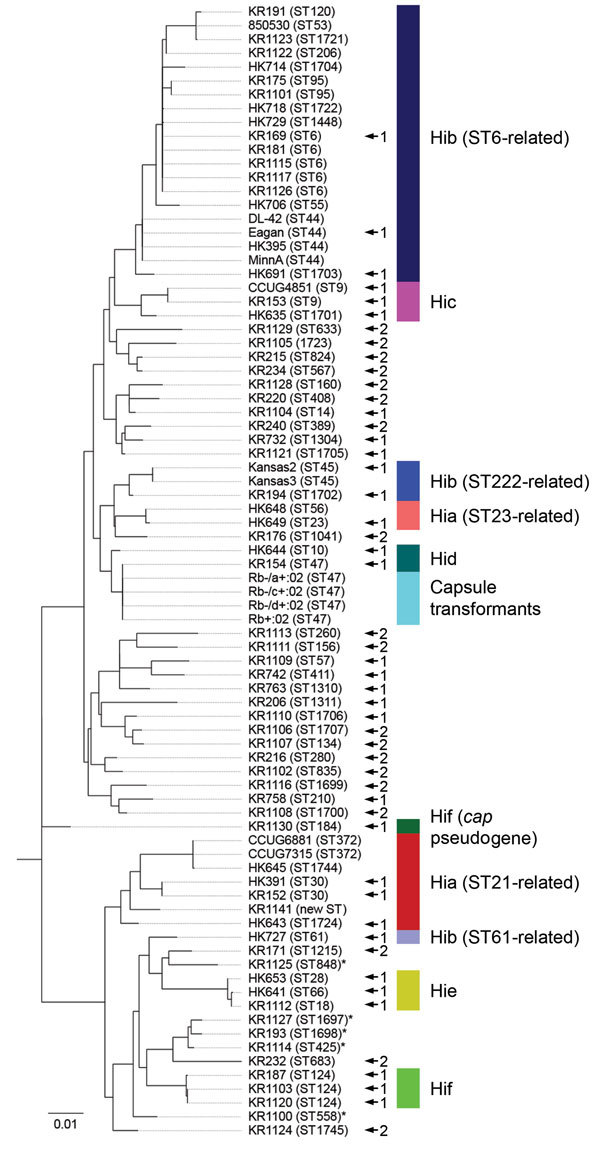
Multilocus sequencing typing (MLST) of encapsulated and nonencapsulated *Haemophilus influenzae* isolates. MLST was performed on a subset of encapsulated isolates (n = 44) from the evaluation set, including all type a and type b isolates (n = 33). All major genetic lineages (indicated by colors), except the least common lineage of Hia (ST4-related), of encapsulated *H. influenzae* were represented in the collection, including the 2 more common lineages of Hia and all 3 lineages of Hib. An isolate typed by PCR as Hif (KR1130) with a nonexpressed pseudogene *cap* locus was also included. This isolate was not part of the established Hif lineage, and was initially suspected to be an outlier on the basis of differences from other Hif in matrix-assisted laser desorption/ionization time-of-flight (MALDI-TOF) mass spectra. All capsule transformants (n = 4) were ST47 (same as the parental strain Rd) (*30**,**31*) and were part of the Hid lineage. Nontypeable *H. influenzae* (NTHi; no color) included as reference isolates in the capsule typing databases (n = 28) were included in the analysis. NTHi in the evaluation set misclassified as type e (n = 5, indicated by asterisks) were also included. These isolates belonged to 3 separate genetic lineages, all related to the Hie lineage. Isolates included as references in MALDI-TOF mass spectrometry databases are indicated by arrows and numbers (1 for isolates in the original database and 2 for isolates added during supplementation of the database). Scale bar indicates nucleotide substitutions per site. Hia, *H. influenzae* type a; Hib, *H. influenzae* type b; Hic, *H. influenzae* type c; Hid, *H. influenzae* type d; Hie, *H. influenzae* type e; Hif, *H. influenzae* type f; ST, sequence type.

To construct a clinically useful reference database for capsule typing by MALDI-TOF mass spectrometry, we aimed to identify and collect isolates from all known lineages of encapsulated *H. influenzae* (Figure 2; online Technical Appendix Table). We performed MLST for all Hia (n = 9) and Hib isolates (n = 24) in the evaluation set, in addition to a subset of isolates of other capsule types (c–f), capsule transformants (n = 4), and NTHi. Phylogenetic analysis confirmed that the collection contained isolates from different genetic lineages of encapsulated *H. influenzae*, including the 2 major genetic groups of type a and all 3 lineages of type b (Figure 2). Capsule transformants belonged to the known genetic lineage of Hid isolates (Figure 2) and were the same ST as the parental strain Rd (*18*). One isolate (KR1130) was typed by PCR as Hif (*bexB*- and *cap f*-positive) but phylogenetically belonged to a lineage separate from all other Hif isolates. Thus, this isolate was not part of the established, ST124-related Hif lineage (Figure 2). The *cap* locus of this isolate was sequenced and found to be a nonexpressed pseudogene (data not shown).

### MALDI-TOF Mass Spectrometry of Genetic Lineages of Encapsulated *H. influenzae*

**Figure 3:**
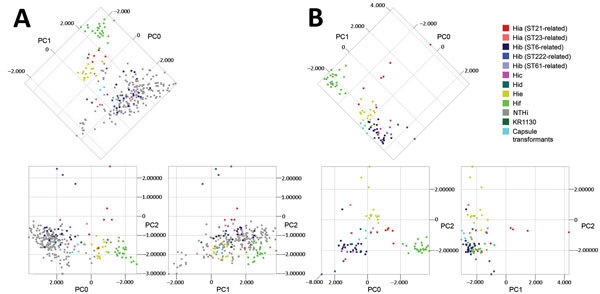
Principal component analysis (PCA) of matrix-assisted laser desorption/ionization time-of-flight mass spectra of encapsulated and nonencapsulated *Haemophilus influenzae*. A) PCA of all isolates (n = 258) of *H. influenzae* in the evaluation set representing all capsule types, which are color-coded according to capsule type and for Hia and Hib isolates by genetic lineage as shown by multilocus sequence typing (MLST) and capsule transformants (n = 4). The first 3 principal components (PC0, PC1, and PC2) are shown in 2-dimensional plots. Analysis showed the diversity of nontypeable *H. influenzae* (NTHi). Encapsulated isolates showed discrete clustering, which was further evaluated by PCA of encapsulated isolates separately. B) PCA of encapsulated isolates in the evaluation set (n = 83) and capsule transformants (n = 4) presented and color-coded as in panel A. Clustering of isolates on the basis of capsule type was evident, particularly for Hib, Hie, and Hif isolates. Different genetic lineages of the same capsule type (Hia and Hib) clustered separately. KR1130 (with a pseudogene type f *cap* locus) did not cluster with the other Hif isolates. Capsule transformants clustered together in proximity of Hid isolates, and not with their respective capsule type. Hia, *H. influenzae* type a; Hib, *H. influenzae* type b; Hic, *H. influenzae* type c; Hid, *H. influenzae* type d; Hie, *H. influenzae* type e; Hif, *H. influenzae* type f; ST, sequence type.

We performed PCA for all isolates in the evaluation set (n = 258) and the capsule transformants (n = 4). As expected, NTHi formed a large heterogeneous group, but clustering of encapsulated isolates of the same capsule types was found (Figure 3, panel A). When PCA was performed on encapsulated isolates (n = 83) and capsule transformants (n = 4) only, the clustering became clearer and was particularly evident for Hib, Hie, and Hif isolates (Figure 3, panel B). Encapsulated isolates segregated in groups according to capsule type and, for Hia and Hib isolates, by genetic lineage according to MLST (Figure 2; 3, panel B). Capsule transformants were found as a separate group in close proximity of Hid isolates and not distributed according to their respective capsule type (Figure 3, panel B). Isolate KR1130 did not cluster with Hif isolates of the ST124-related lineage (Figure 3, panel B).

**Figure 4:**
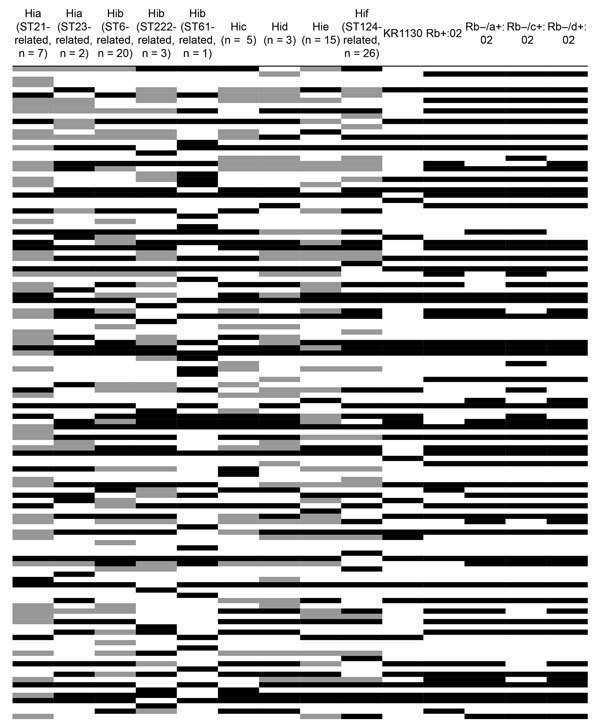
Biomarker analysis of matrix-assisted laser desorption/ionization time-of-flight mass spectra of encapsulated *Haemophilus influenzae*. Analysis was performed on all encapsulated isolates in the evaluation set (n = 83) and capsule transformants (n = 4). Rows represent peaks (2,000–20,000 m/z in descending order), and columns represent groups of encapsulated *H. influenzae*. A total of 124 peaks with discriminatory power (p<0.05) between different capsule types and genetic lineages were identified. Peak expression is indicated by shades of black (black >75%, gray >25% but <75%, and white <25% of isolates in the group express the peak). Several peaks conserved within capsule types and genetic lineages with the possibility for separation were observed, as indicated by the mosaic of peak patterns. Capsule transformants showed similar peak patterns, and lacked many of the capsule type-specific peaks for their respective phenotypic capsule types. KR1130 expressed different peaks than Hif of the ST124-related lineage. Hia, *H. influenzae* type a; Hib, *H. influenzae* type b; Hic, *H. influenzae* type c; Hid, *H. influenzae* type d; Hie, *H. influenzae* type e; Hif, *H. influenzae* type f; ST, sequence type.

Biomarker analysis of encapsulated isolates and capsule transformants identified several peaks conserved within the different genetic lineages of capsule types, indicating the possibility of separating them on the basis of MALDI-TOF mass spectra (Figure 4). Capsule transformants expressed similar peak patterns relative to each other but differed in many peaks when compared with wild-type strains of the same capsule types.

### Sensitivity and Specificity of Automated Capsule Typing by MALDI-TOF Mass Spectrometry

An initial capsule typing reference database was constructed in MALDI Biotyper. Encapsulated isolates (n = 22) representing all major genetic lineages of encapsulated *H. influenzae* were included (Figure 2). To ensure adequate coverage of potential variation within each lineage, multiple reference isolates were chosen for each lineage (when possible) on the basis of geographic origin and variations in mass spectra. In addition, NTHi (n = 9) representing 8 of 10 known genetic clades of NTHi (*19*) were included in the database (Figure 2).

Validation of the original database (n = 31) using the remaining isolates in the evaluation set (n = 227) showed 100% sensitivity for encapsulated isolates (Table 1), and every isolate was correctly classified on every spectrum. All capsule transformants were classified as type d, the original serotype of the parental strain Rd (*31*). No isolate matched KR1130, the isolate typed by PCR as Hif with a pseudogene *cap* locus.

**Table 1:** Validation of the original MALDI-TOF mass spectrometry capsule typing database (n = 31) by classification of the remaining 227 isolates in the evaluation set and 4 capsule transformants of Haemophilus influenzae*

Capsule type	No.	No. correct†	No. inconclusive‡	No. incorrect§	Sensitivity, %	Specificity, %
Hia, ST21-related	4	4	0	0	100	99.1
Hia, ST23-related	1	1	0	0	100	100
Hib, ST6-related	17	17	0	0	100	100
Hib, ST222-related	1	1	0	0	100	100
Hic	2	2	0	0	100	100
Hid	1	1	0	0	100	98.2
Hie	12	12	0	0	100	97.7
Hif	23	23¶	0	0	100	99.0
All encapsulated isolates, a–f	61	61	0	0	100	92.2
Nontypeable	166	122	31	13#	73.5	100
Rb-negative capsule transformants	4	1**	0	3**	NA	NA

*Hia, *H. influenzae* type a; Hib, *H. influenzae* type b; Hic, *H. influenzae* type c; Hid, *H. influenzae* type d; Hie, *H. influenzae* type e; Hif, *H. influenzae* type f; M ALDI-TOF, matrix-assisted laser desorption/ionization time-of-flight; NA, not applicable; NTHi, nontypeable *H. Influenzae*; ST, sequence type. †>5/6 spectra classified to the same correct type. ‡<4/6 spectra classified to the same type. §>5/6 spectra classified to the same incorrect type. ¶All classified to the established ST124-related Hif lineage. #Two isolates classified as Hia (ST21-related), 4 as Hid, 5 as Hie, and, 2 as Hif (ST124-related). **All classified as type d (i.e., the same type as the parental strain Rd) (*30**,**31*), resulting in correct classification of isolate Rb–/d+:02 and incorrect classification of isolates Rb+:02, Rb–/a+:02, and Rb–/c+:02.

**Figure 5:**
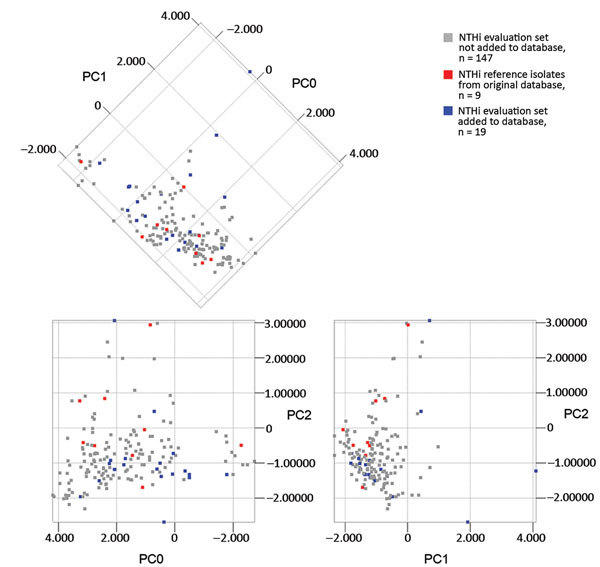
Principal component analysis of matrix-assisted laser desorption/ionization time-of-flight mass spectra of NTHi in the original and supplemented databases. Isolates are color-coded according to database affiliation, and the first 3 principal components (PC0, PC1, and PC2) are shown in 2-dimensional plots. No clustering similar to that for encapsulated isolates was observed. NTHi reference isolates in the original capsule typing database (n = 9), representing different genetic clades, were evenly distributed in the group. Supplementing the reference database with another 19 isolates improved coverage of the heterogeneity of NTHi. NTHi, nontypeable *H. influenzae*; PCA, principal component analysis.

A few NTHi were either inconclusively typed or misclassified as encapsulated, resulting in reduced specificity for encapsulated isolates (Table 1). For this reason, we supplemented the capsule typing database with misclassified NTHi from the evaluation set until the database correctly classified all the remaining isolates in the evaluation set on every single spectrum. This modification resulted in an additional 19 NTHi being added to the MALDI Biotyper database (Figure 2). When PCA was performed separately for NTHi in the evaluation set, it was evident that the supplemented database covered the heterogeneity of NTHi better than the original database (Figure 5). The same finding was evident from phylogenetic analysis (Figure 2). Five NTHi were misclassified as Hie and could not be added to the database because they interfered with classification of true Hie isolates and would decrease sensitivity for Hie (Figure 2).

As a final performance test, we blindly validated the supplemented database (n = 50) by using a separate culture collection consisting of clinical invasive isolates from Sweden (n = 126) obtained during 2010 and 2013–2016. When we compared MALDI-TOF mass spectrometry capsule typing results with PCR capsule typing results, all encapsulated isolates (types b, e, and f; n = 28) were correctly classified on every single spectrum (Table 2). Of 98 NTHi, only 5 were not correctly classified. These isolates were all classified as inconclusive. Thus, no NTHi was incorrectly classified as encapsulated, and the resulting sensitivity and specificity of capsule typing was 100% in the final validation (Table 2).

**Table 2:** Validation of the supplemented MALDI-TOF mass spectrometry capsule typing database (n = 50) by classification of 126 invasive isolates of *Haemophilus influenzae* from Sweden*

Capsule type	No. tested	No. correct†	No inconclusive‡	No. incorrect§	Sensitivity, %	Specificity, %
Hib	8	8¶	0	0	100	100
Hie	5	5	0	0	100	100
Hif	15	15#	0	0	100	100
All encapsulated isolates (b, e, and f)	28	28	0	0	100	100
NTHi	98	93	5	0	94.9	100

*Hib, *H. influenzae* type b; Hie, *H. influenzae* type e; Hif, *H. influenza* type f; MALDI-TOF, matrix-assisted laser desorption/ionization time-of-flight; ST, sequence type. †>5/6 spectra classified to the same correct type. ‡<4/6 spectra classified to the same type. §>5/6 spectra classified to the same incorrect type. ¶All classified to the ST6-related Hib lineage. #All classified to the established ST124-related Hif lineage.

## Discussion

In this study, we have shown that encapsulated *H. influenzae* have different MALDI-TOF mass spectra that correlate with genetic lineages representing different capsule types. We have demonstrated that, after construction of a comprehensive reference database, routine MALDI-TOF mass spectrometry analysis has excellent capacity for identifying type-specific genetic lineages associated with encapsulated *H. influenzae* and thereby can be used for capsule typing of *H. influenzae*.

Our study had several strengths. We analyzed a large collection of well-characterized strains collected at different times from various geographic regions to ensure the robustness of our findings. Using MLST, we ensured adequate coverage of the major genetic lineages of encapsulated *H. influenzae* in the MALDI-TOF mass spectrometry reference database. Moreover, the database was carefully evaluated and supplemented to ensure adequate coverage of the heterogeneity of NTHi. We blindly validated the supplemented database to mimic an authentic clinical or epidemiologic situation and demonstrated excellent sensitivity and specificity compared with conventional PCR-based typing. During construction of the capsule typing database, we identified several isolates previously typed by PCR or agglutination (by us or others) in which the MALDI-TOF mass spectrometry results did not match the suggested capsule type. When we retyped these isolates by PCR, the capsule type suggested by MALDI-TOF mass spectrometry proved to be correct in all instances (except for the NTHi typed as Hie) (Figure 2), and isolates were reassigned to a new capsule type, further supporting the capacity of MALDI-TOF mass spectrometry for capsule typing.

Our study had some limitations. The first limitation reflects the limited availability of some rare variants. Our collection contained no Hia isolates belonging to the uncommon ST4-related genetic group. For the ST61-related lineage of Hib, we had access to only 1 isolate, which was included in the reference database and thus not represented in the test collection. However, our ST61 isolate was separable when mass spectra were analyzed by PCA and biomarker analysis, and no isolate was misclassified to this lineage in the initial or final validation of the typing databases. Furthermore, we have demonstrated that identification of the ST222-related Hib lineage by MALDI-TOF mass spectrometry is possible (Table 1), which was not the case previously (*29*). The second potential limitation arises through the genetic heterogeneity of NTHi, making adequate representation in the reference database a challenge (*19*). This limitation was apparent during the initial evaluation of the typing method, when some NTHi were misclassified. To address this issue, we supplemented the database with 19 additional reference NTHi strains. The final validation of our typing method demonstrated excellent specificity for NTHi, but the sensitivity for identifying encapsulated isolates remained unchanged. Because most invasive infections in countries implementing Hib vaccination are caused by NTHi, a high specificity is desirable (*3**–**5*).

MALDI-TOF mass spectrometry has proved valuable in subtyping several clinically relevant bacteria, including *Clostridium difficile* (*36*), methicillin-resistant *Staphylococcus aureus* (*37**,**38*), and enterohemorrhagic *Escherichia coli* (*39*). Subtyping generally relies on common genetic differences between isolates, reflected in the composition of the proteins measured. In our study, wild-type isolates of different capsule types could be separated, but isogenic capsule transformants could not. These isolates were classified as type d, the original capsule type of the parental strain Rd. This finding confirms that capsule type identification is based on a proxy identification of genetic lineage, rather than identification of capsule biosynthesis-associated proteins. Thus, our method is an indirect typing method, as opposed to serotyping, which identifies the capsule polysaccharide, and PCR, which identifies the capsule gene complex directly.

Although there is little evidence that new lineages of encapsulated *H. influenzae* have appeared historically, novel lineages of encapsulated strains might appear and be missed by the method. Isolate KR1130 used in this study was initially suspected to represent such a lineage. However, its *cap* locus was shown to be on a nonexpressed pseudogene. Only 1 other isolate of the same ST (ST184) is currently registered in the MLST database, and it is a nontypeable isolate. No other Hif strain in this study or the MLST database belongs to this genetic lineage (*20*).

One advantage of indirect capsule type identification by MALDI-TOF mass spectrometry is that determination of genetic lineage of encapsulated isolates can be made without further analysis. The method can also identify previously encapsulated capsule-deficient strains, which have lost parts or all of the *cap* locus, either during infection or laboratory handling (*40**–**42*).

A concern regarding subtyping by MALDI-TOF mass spectrometry (*43*) is the potential need for special sample preparations, such as growth conditions and type of matrix. In several studies, differences in mass spectra between subtypes of various species were observed but no automated classification methods were reported (*43*), which might limit general applicability. In this study, we used standard growth conditions, as well as routine ethanol–formic acid extraction and mass spectra acquisition protocols. The software used (MALDI Biotyper) also has the advantage of being a standard software used in clinical settings. These factors greatly increased the chance of clinical implementation of our findings.

In conclusion, our study demonstrated that rapid capsule typing of *H. influenzae* by identification of capsule type-specific genetic lineages using routine MALDI-TOF mass spectrometry is possible and highly accurate. After further large-scale validation, this method has the potential for clinical and research use. With the increasing heterogeneity in capsule types of disease-causing *H. influenzae* observed since Hib conjugate vaccines were introduced, the method can become a valuable tool in clinical diagnostic laboratories.

Technical Appendix Additional information on capsule typing of *Haemophilus influenzae* by matrix-assisted laser desorption/ionization time-of-flight mass spectrometry.
