# Supplementary material for: Capsule Typing of Haemophilus influenzae by Matrix-Assisted Laser Desorption/Ionization Time-of-Flight Mass Spectrometry
Source: Emerg Infect Dis. 2018 Mar;24(3):443–52. doi: 10.3201/eid2403.170459 (PMC5823330; doi:10.3201/eid2403.170459)
Supplement: Technical Appendix — Additional information on capsule typing of Haemophilus influenzae by matrix-assisted laser desorption/ionization time-of-flight mass spectrometry. [file 17-0459-Techapp-s1.pdf]

Article DOI: https://doi.org/10.3201/eid2403.170459

Capsule Typing of *Haemophilus influenzae* by Matrix-Assisted Laser Desorption/Ionization Time-of-Flight Mass Spectrometry

Technical Appendix

Technical Appendix Table. Isolates of *Haemophilus influenzae* used to develop a MALDI-TOF mass spectrometry typing database*

Isolate	Other designation	Capsule type	Location	Year	Tissue/infection
International					
CCUG6881	Smith	a	Unknown	Before 1973	Unknown
CCUG7315	NCTC8465	a	USA	1941	Respiratory tract
HK391	ATCC9327, NCTC8466	a	USA	1942	CSF/nasal secretion
HK643	Fin31	a	Finland	1974–1976	Respiratory tract
HK645	Harding	a	Boston, MA, USA	1981	CSF
HK648	CDC78, E202	a	Unknown	Unknown	Unknown
HK649	CDC22, CDC D5361	a	USA	1976	Blood
KR152		a	Sweden	Unknown	Unknown
KR1141		a	Angola	2016	Ear swab
DL-42		b	Dallas, TX, USA	Before 1984	Unknown
Eagan	CCUG18095	b	Boston, MA, USA	1968	CSF
HK395		b	USA	Before 1954	Unknown
HK691	A1132st	b	USA	Before 1985	Unknown
HK706	M1053	b	USA	1979	Unknown
HK714	M1062	b	USA	1980	Unknown
HK718	M1071	b	USA	1941	Unknown
HK727	M1084	b	USA	1947	CSF
HK729	M1077	b	USA	1980	CSF
Kansas2		b	Missouri, USA	2014	Epiglottitis
Kansas3		b	Missouri, USA	2014	Osteomyelitis
MinnA		b	Minneapolis, MN, USA	1979	CSF
850530		b	The Netherlands	1985	Unknown
CCUG4851	NCTC8469, ATCC9007	c	USA	1942	Sputum
CCUG4852	Ruggerio	c	New York, NY, USA	Before 1950	CSF
HK635	43/LA	c	Papua New Guinea	1980	Lung aspirate
HK688		c	Copenhagen, Denmark	1984	CSF
KR153		c	Sweden	Unknown	Unknown
HK644	51/LA	d	Papua New Guinea	1979	Unknown
KR154		d	Sweden	Unknown	Unknown
NCTC8470		d	The Netherlands	1937	Respiratory tract
CCUG15521		e	Probably Sweden	Before 1984	Unknown
KR1142		e	Unknown	Unknown	Unknown
HK636	CDC54	e	Unknown	Unknown	Unknown
HK641	Hepke	e	Denver, CO, USA	1980	Unknown
HK653	45/LA	e	Papua New Guinea	1979	Unknown
KR138	A76/01	e	Unknown	Unknown	Unknown
KR147	A77/99	e	Unknown	Unknown	Unknown
CCUG15435	Shawn	f	Unknown	Unknown	Unknown
CCUG6877		NT	USA	1941	Unknown
CCUG15519		NT	Unknown	Before 2001	Unknown
HK224		NT	Denmark	Before 1976	Otitis media
Sweden					
11		b	Gothenburg, Lund/Malmö, Stockholm	1998–2007	Blood, CSF
8		e	Lund/Malmö, Stockholm	2006–2009	Blood, CSF, respiratory tract

Isolate	Other designation	Capsule type	Location	Year	Tissue/infection
26		f	Gothenburg, Lund/Malmö, Stockholm	1997–2009, 2011	Blood, CSF
172		NT	Gothenburg, Lund/Malmö, Stockholm	1997–2011	Blood, CSF, respiratory tract

*ATCC, American Type Culture Collection; CCUG, Culture Collection of the University of Göteborg; CDC; US Centers for Disease Control and Prevention; CSF, cerebrospinal fluid; MALDI-TOF, matrix-assisted laser desorption/ionization time-of-flight; NCTC, National Collection of Type Cultures; NT, nontypeable.
